# Perforin Expression by CD4+ Regulatory T Cells Increases at Multiple Sclerosis Relapse: Sex Differences

**DOI:** 10.3390/ijms13066698

**Published:** 2012-06-01

**Authors:** Marta Tejera-Alhambra, Bárbara Alonso, Roseta Teijeiro, Rocío Ramos-Medina, Carol Aristimuño, Larissa Valor, Clara de Andrés, Silvia Sánchez-Ramón

**Affiliations:** 1 Department of Immunology, Hospital General Universitario Gregorio Marañón, Doctor Esquerdo 46, Madrid 28007, Spain; E-Mails: marta.tejeraal@salud.madrid.org (M.T.-A.); barbara.alonso@hotmail.com (B.A.); rosetatm@yahoo.es (R.T.); rrm-83@hotmail.com (R.R.-M.); aristimuno@gaiker.es (C.A.); valormendez@yahoo.de (L.V.); 2 Department of Neurology, General University Hospital Gregorio Marañón, Doctor Esquerdo 46, Madrid 28007, Spain; E-Mail: claradeandres@hotmail.com

**Keywords:** cerebrospinal fluid, regulatory T-lymphocytes, multiple sclerosis, perforin expression

## Abstract

Multiple sclerosis (MS) represents the leading cause of neurological deficit among young adults, affecting women more frequently than men. In MS, the extent of central nervous system lesions is determined by the net balance between self-reactive and regulatory T-cells at any given time, among other factors, as well as by the effect of inflammatory response. Here, we studied both CD4+ and CD8+ T_Reg_ in parallel in blood and CSF during MS relapse. A recruitment of both regulatory CD4+ and CD8+ T cells (T_Reg_) within the cerebrospinal fluid (CSF) takes place during MS relapse. Not previously described, the presence of CD4+ T_Reg_ in CSF was higher in women than in men, which could account for the sexual dimorphism in the incidence of MS. A direct correlation between plasma oestradiol (E2) and IL-2 levels was observed, in line with a putative circuit of E2 and perforin expression by CD4+ T_Reg_ playing a role in MS. Also, serum IFN-alpha was higher in females, with direct correlation with serum E2 levels. This is the first study to analyze perforin expression by CD4+ T_Reg_ in MS, which was greatly enhanced in CSF, what points out a relevant role of this molecule in the suppressive effects of the CD4+ T_Reg_ in MS, and contributes to the understanding of MS pathophysiology.

## 1. Introduction

Multiple sclerosis (MS), a prototype autoimmune disease of the central nervous system (CNS), is the leading cause of neurological deficit among Caucasian young adults [1]. MS is a chronic inflammatory demyelinating disorder, in which the interplay of diverse self-reactive and regulatory T cell subsets drives the magnitude of tissue damage and the duration of MS relapse. Theoretically, a disturbed balance between cells that induce or cause demyelination (self-reactive effector T-cells, mainly Th1 and Th17) and regulatory CD4+ and CD8+ T cells (T_Reg_) that are capable of suppressing these self-reactive T cells underlies MS pathogenesis [2].

CD4+ T_Reg_ play an important role in maintaining immune homeostasis and thus in preventing autoimmune diseases, such as MS. Indeed, T_Reg_ from patients with relapsing-remitting MS (RR-MS) have shown a diminished suppressive function [3–5]. Several subsets of regulatory T-cells have been already identified in mice and humans [6], from which the CD4+CD25^hi+^FoxP3+ T_Reg_ subset exhibits the strongest suppressive function [7]. On the other hand, the CD8+ regulatory/suppressive T-cell subsets although were the first to be described [8] have not been more thoroughly characterized until recently [9]. Similarly to CD4+ T_Reg_, CD8+ T_Reg_ mechanisms for suppressing immune responses involve cell-to-cell contact-mediated suppression and anti-inflammatory cytokines’ secretion (IL-10 and TGF-β) [10]. Different lines of evidence suggest that CD8+ T_Reg_ are a highly heterogeneous cell population that contributes to the suppression of immune responses by different mechanisms not fully understood. We and others have studied the presence and function of circulating CD4+ and CD8+ T_Reg_ of patients with MS [3–5,11–13]. However, the presence of such T_Reg_ subsets within the CNS compartment of MS patients has been studied in a very few studies [14–16]. The cerebrospinal fluid (CSF) seems to be an adequate compartment to analyze the immunological signature reflecting the actual inflammation within the CNS, due to the difficulty of studying the brain, the target organ in MS disease. In this observational study, we studied the presence of CD4+ and CD8+ T_Reg_ lymphocytes in the CSF and peripheral blood. As one of the recently proposed suppressive mechanisms of human CD4+ T_Reg_ that causes autologous target cell death in cancer and autoimmunity involves the perforin-granzyme pathway [11,17,18], we sought to ascertain whether the expression of perforin by these regulatory lymphocytes was a relevant mechanism in MS pathophysiology.

On the other hand, a sexual dimorphism exists in the incidence and severity of autoimmune diseases, such as MS, which seems to be intrinsically involved in the pathophysiology of these diseases. In fact, MS is twice as frequent in women than in men [19], with clinical onset at reproductive age and clinical variations with menstrual cycle and pregnancy. Our group has demonstrated that oestradiol (E2) enhances the suppressive function of CD4+ T_Reg_ *in vitro*, process mediated in part by perforin [17]. For this reason, we also sought to determine the contribution of CD4+ and CD8+ T_Reg_ both in women and men within the CNS compartment at MS relapse. This approach has not previously been explored.

## 2. Results and Discussion

### 2.1. Comparison among Proportions of Regulatory CD4+ and CD8+ T-Cells Subsets in CSF and Peripheral Blood in MS Patients at First Clinical Relapse

**Figure 1:**
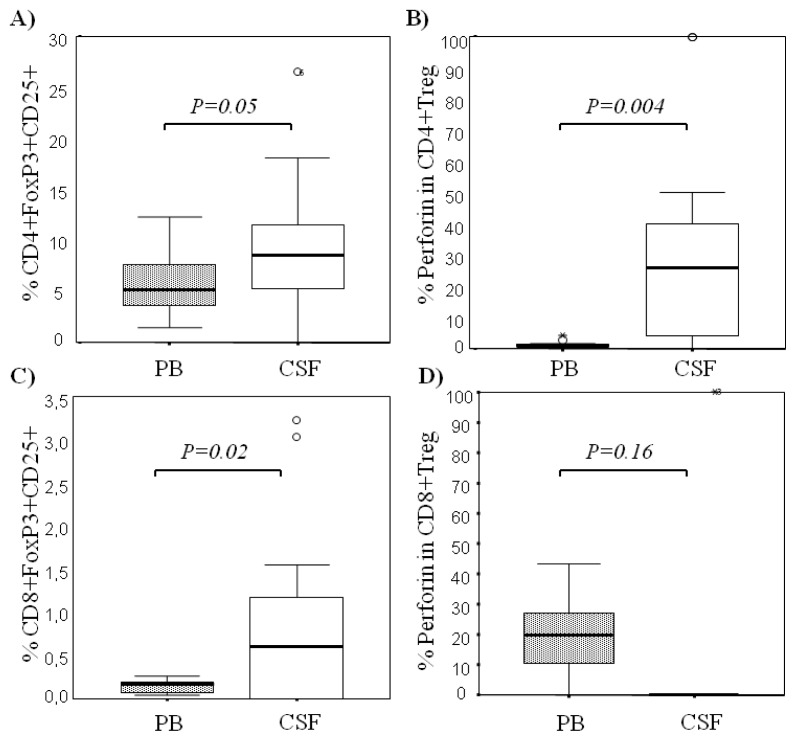
CD4+ T_Reg_ and CD8+ T_Reg_ frequencies with their expression of perforin in the cerebrospinal fluid (CSF) and in peripheral blood (PB) of MS patients with first clinical isolated syndrome (CIS). (**A**) CD4+ T_Reg_, defined as CD4+FoxP3+CD25+, frequencies in CSF and PB; (**B**) CD4+ T_Reg_ expression of perforin in CSF and PB; (**C**) CD8+ T_Reg_, defined as CD8+FoxP3+CD25+ frequencies in CSF and PB; (**D**) CD8+ T_Reg_ expression of perforin in CSF and PB.

When we studied paired samples of peripheral blood and CSF in MS patients at first clinical relapse, the frequencies of CD4+ T_Reg_ (CD4+CD25+FoxP3+ and CD4+CD25bright) were significantly higher in CSF in comparison to their peripheral blood counterparts: CD4+CD25+FoxP3+ were 8.96 ± 6.84, 8.49 (7.89) *vs*. 5.65 ± 2.90, 5.13 (4.55), (*p* = 0.053) and CD4+CD25bright were 3.19 ± 1.77, 3.23 (2.91) *vs*. 1.23 ± 0.57, 1.20 (0.91) (*p* = 0.002), respectively. The proportions of CD4+CD25+FoxP3+ in CSF and PB are shown in Figure 1A. Notably, intracellular expression of perforin on CD4+ T_Reg_ was significantly higher in CSF than in circulating cells: The percentages of CD4+CD25+FoxP3+ T_Reg_ that co-expressed perforin in the CSF compartment were 32.32 ± 32.65, 25.82 (39.37) *vs*. 1.54 ± 1.22, 1.24 (1.4), *p* = 0.004 (Figure 1B). At the single cell level, the mean fluorescence intensity (MFI) of perforin in CD4+ T_Reg_ showed a trend to be higher in the CSF than in the periphery: 79.60 ± 87.06, 52.77 (132.95) *vs*. 45.50 ± 47.10, 26.64 (45.81), *p* = 0.1.

The MFI of the co-stimulatory molecule CD28 on CD4+ T_Reg_ was also analyzed in a subgroup of patients (*n* = 10) and interestingly, we found that the expression of this co-stimulatory signal was significantly reduced in CD4+ T_Reg_ in the CSF compared to the periphery (MFI CD4+CD25^med^: 69.71 ± 40.76 *vs*. 289.75 ± 63.87, *p* < 0.0001; MFI CD4+CD25^hi+^: 79.08 ± 40.28 *vs*. 325.77 ± 68.01, *p* = 0.006).

With respect to CD8+ T_Reg_ subsets (CD8+FoxP3+, CD8+CD25+FoxP3+) were also significantly higher in the CSF compartment with respect to the periphery (Table 1). Most of the CD8+FoxP3+ T_Reg_ expressed on their surface the molecule CD25 and both subsets, CD8+FoxP3+ and CD8+CD25+FoxP3+ (Figure 1C), were present in higher proportions in CSF (*p* = 0.02 and *p* = 0.02, respectively) than in peripheral blood. The proportions of CD8+ T_Reg_ are illustrated in Table 1. In contrast to CD4+ T_Reg_, most of the CD8+CD25+FoxP3+ in CSF did not coexpress perforin (Median 0 (0) *vs*. 21.47 (22.73)) (Figure 1D).

### 2.2. Comparison of Regulatory T-Cells and Their Content of Perforin in Peripheral Blood between MS Patients and Healthy Controls

We found no differences in the frequencies of CD4+CD25+FoxP3+ T_Reg_ between MS patients at MS relapse and healthy controls. CD4+CD25^hi+^ T_Reg_ showed a trend to a higher frequency in healthy controls than in MS patients at relapse 1.27 ± 0.73, 1.24 (1.03) *vs*. 0.93 ± 0.64, 1.01 (1.22) *p* = 0.08. When comparing only women, the differences reached statistical significance (1.30 ± 0.70, 1.26 (0.99) *vs*. 0.84 ± 0.68, 0.73 (1.27) *p* = 0.03). By contrast, there were no such differences between healthy controls and patients in any of the CD8+ T_Reg_ subsets studied.

MS is associated with a functional defect of natural CD4+ T_Reg_ [4,5,20], defect that can be restored by some disease course modifying therapies, such as IFNβ or copaxone [3]. This study simultaneously analyzed CD4+ and CD8+ T_Reg_ in CSF and peripheral blood, confirming the higher frequencies of CD4+CD25+FoxP3+ T_Reg_ in the CSF compared to the blood in a series of patients at first clinical relapse that were later on diagnosed as MS, in line with authors that had characterized CSF T_Reg_ as CD4+CD25^hi+^CD27+ [14,21] and as CD4+CD25+FoxP3+ [15]. This is the first study analyzing the perforin expression on T_Reg_ within the CSF during MS relapse, pointing out this molecule as a putative mechanism mediating suppressive activity of CD4+ T_Reg_ at the CNS, but not by CD8+ T_Reg_. In contrast, negligible quantities of perforin were expressed by their circulating CD4+ T_Reg_ counterparts. Prior studies on cancer patients and in the mice model had shown that perforin expression on CD4+ T_Reg_ was a relevant cell-cell contact suppression mechanism of this cell subset [18,22], further indicating that perforin expression could be mediating direct killing of dendritic cells and other target cells. A recent study has shown that immune regulation mediated by T_Reg_ is dependent on the presence of IFN-γ and the perforin pathway to suppress EAE, the animal model of MS [23].

Our group has recently described an E2-dependent perforin expression on CD4+ T_Reg_ of healthy pregnants [17]. In the present study, we show that this perforin-dependent mechanism may play a relevant role in MS. Indeed, the proportions and the expression of perforin at the single cell level in CD4+ T_Reg_ in the CSF were significantly higher than those in peripheral blood. We have previously demonstrated the expression of surface lysosomal-associated membrane glycoproteins (LAMP)-1 and LAMP-2 by *in vitro* activated T_Reg_, which is a sign of cell degranulation and of the cytotoxicity exerted by these cells [17]. Therefore, such enhanced perforin expression by CD4+ T_Reg_ within the CSF of MS patients at first clinical relapse represents the first *in vivo* report indicating that perforin might play a role in the cytotoxicity and killing of target T cells, namely autorreactive/effector cells, in order to counteract the damage by autoreactive T and B cells. Indeed, a recent work shows that perforin involves a molecular mechanism of T_Reg_-mediated killing of the pathogenic T cells in the experimental model of MS [23]. Also, in a tumor mice model, perforin mediated a tumor-antigen-dependent mechanism of DC killing by T_Reg_ [22]. Other data point out a relevant role of perforin in the pathogenesis of MS. Variations in the PRF1 gene have been described in MS patients with a higher frequency than in healthy controls [24]. Other authors have described a generalized defect in the expression of perforin in a subgroup of male patients with PPMS [25]. By contrast, CD8+ T_Reg_ content of perforin was significantly decreased in the CNS as compared to blood, which suggests that this subset does not seem to use the perforin pathway for their suppressive function in MS within the central nervous compartment. In this study, we found also higher frequencies of CD8+CD25+FoxP3+ T_Reg_ within the CNS compartment. This is in contrast to other authors that have recently described lower levels of CD8+CD25+FoxP3+ T_Reg_ in the CSF than in blood of patients with MS during acute exacerbations [16].

On the other hand, CD28 expression was markedly and significantly diminished on CD4+ T lymphocytes in the CSF of patients at acute MS relapse compared with that at the peripheral blood. CD28 is constitutively expressed on the surface of naïve T cells and its role in co-stimulation has been demonstrated in a variety of model systems [26]. This finding points out an additional immune mechanism of peripheral tolerance taking place as part of the immune response within the CNS compartment.

### 2.3. Sexual Dimorphism in Regulatory T Cells

**Figure 2:**
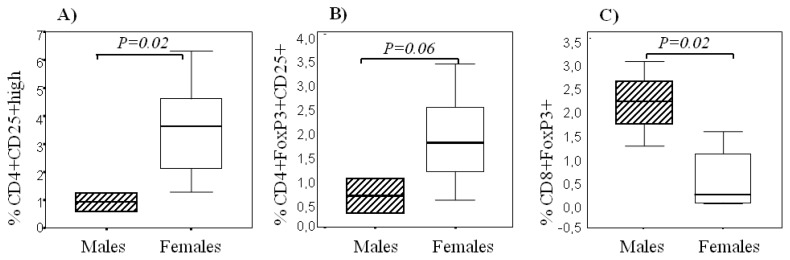
Sexual dimorphism in regulatory T cells in the cerebrospinal fluid (CSF) of MS patients with first clinical isolated syndrome (CIS). (**A**) CD4+ T_Reg_, defined as CD4+CD25^hi+^, frequencies in the CSF of men and women with CIS; (**B**) CD4+ T_Reg_, defined as CD4+FoxP3+CD25+; and (**C**) CD8+ T_Reg_, defined as CD8+ FoxP3+, frequencies in the CSF of men and women with CIS.

We sought to determine the contribution of CD4+ and CD8+ T_Reg_ in both women and men within CNS compartment at MS relapse. Our results pointed to something that has not been previously described: females presented higher frequencies of CD4+ T_Reg_, namely CD4+CD25^hi+^ (*p* = 0.02) (Figure 2A), CD4+CD25+FoxP3+ (*p* = 0.06) (Figure 2B) in the CSF compartment. By contrast, males presented significantly higher proportions of CD8+ T_Reg_, measured as CD8+FoxP3**+** (*p* = 0.02) (Figure 2C). In the peripheral blood of MS patients, we observed again a trend of higher proportions of CD4+CD25+FoxP3+ T_Reg_ in females than in males (*p* = 0.1). By contrast, CD4+CD25+FoxP3+ T_Reg_ in the healthy control group were more abundant in men than in all women (*p* = 0.01). However, when we compared healthy women at day 14 of the menstrual cycle and men, females’ CD4+CD25+FoxP3+ T_Reg_ co-expressed significantly higher perforin levels than males (median in females, 0.84 *vs*. median in males, 0.0, *p* = 0.004) and MFI of perforin (median in females, 20.06 *vs*. median in males, 0.0, *p* < 0.001). A direct correlation was observed between the percentage of perforin in CD4+CD25+FoxP3+ T_Reg_ and the levels of serum testosterone in women from the group of healthy controls (*r* = 0.34, *p* = 0.01). Moreover, in healthy controls the percentage of CD4+ T_Reg_ correlated with the levels of serum testosterone both in men (*r* = 0.52, *p* = 0.05) and in women (*r* = 0.45, *p* < 0.0001).

Compatible with the data previously reported by us for perforin expression on T_Reg_ in the setting of normal pregnancy, expression of perforin in CSF of MS patients was confined to the FoxP3+ and HLA-DR+ T_Reg_ subsets. All T_Reg_ expressing perforin were also positive for granzyme (data not shown).

The presence of CD4+CD25^hi+^ was higher within the CSF in MS females with respect to MS males and the presence of CD4+ T_Reg_ within the CSF showed a trend to be higher, whereas CD8+ T_Reg_ lymphocytes within CSF were higher in males. This has not been previously described, Recent results of our group have shown that E2 *in vitro* enhances the percentages and suppressive function of CD4+ T_Reg_ [17]; and that CD4+ T_Reg_ show a differential higher expression of E2 and progesterone receptors than effector T-cells, which may render CD4+ T_Reg_ more sensitive to these sex hormones [27]. Our findings in this cohort of MS patients show that perforin expression on CD4+ T_Reg_ was higher in MS males. This could be due to the diluting effect of MS women at any time point of the menstrual cycle and to the activation of the hypothalamic-pituitary-gonadal axis during MS relapse. However, when we compared the group of healthy women at day 14 with healthy men, expression of perforin on CD4+ T_Reg_ was higher in women, in line with our previous results [17]. These studies suggest an important role of these lymphocytes subset in the regulation of the effector response in the CNS, which could contribute to the understanding of why women are more prone to MS relapses than men, while the disease in men tends to be more severe. All this body of evidence suggests that perforin may play a relevant role in the pathogenesis of MS and gender might affect its expression. A defective perforin cell-mediated suppression on CD4+ T_Reg_ may thus have a role in lymphocyte accumulation and autoimmune disease.

### 2.4. Sex Hormones and Cytokine Levels

The content of sex hormones in the CSF and in plasma samples (*n* = 17) was analyzed. As expected, the content of measurable sex hormones in the CSF compartment was negligible. Progesterone was only detected in three CSF samples of three women (0.2, 0.3, 0.5 μg/L). Oestradiol (E2) was not detected in any of the CSF samples analyzed. Testosterone was only detected in one of the five males analyzed (0.2 μg/L). By contrast, the stress hormone cortisol was detected in the CSF samples from 7 out of the 12 women analyzed and in all the 5 men analyzed (median (IQR), 1.2 (0.2) μg/dL). We found no differences by sex in the concentration of cortisol.

From the set of 11 cytokines and chemokines measured in parallel in blood and CSF (pairs of CSF and plasma from 10 MS patients, 5 males and 5 females), only IL-2 and IFN-α were detected in the CSF of our group of MS patients at relapse: IL-2 (*n* = 7) (median (IQR), 22.82 (0.94); IFN-α (*n* = 9) 17.67, (3.97). In plasma, the following cytokines were detected: IL12p70 (*n* = 3) 6.54, (5.29); IL-2 (*n* = 9) 28.42, (5.25); IL10 (*n* = 2) 7.62, (1.71); IFN-α (*n* = 9) 22.37, (2.48); TNF-α (*n* = 8) 18.28, (2.30); RANTES (*n* = 10) 2,652.11, (3346.89). All the cytokines were measured in pg/mL. Serum IFN-α was higher in women than in men (mean ± SD, median (IQR), women 27.13 ± 6.64, 23.7 (12.67); men 17.50 ± 9.82, 21.22 (12.2), *p* = 0.1), though this difference was not statistically significant probably due to the small number of samples.

Interestingly, a positive correlation was found between serum E2 and IL-2 showed (*r* = 0.64, *p* = 0.04); and a trend between E2 and IFN-α (*r* = 0.57, *p* = 0.08), whilst with testosterone there was a trend of inverse correlation (*r* = −0.57, *p* = 0.08).

When we analyzed the concentration of key cytokines and sex hormones in pairwise plasma and CSF samples IFN-α and IL-2 were the only cytokines detected both in plasma and CSF. TNF-α and RANTES were only detected in plasma. IL-2 has been described to modulate *in vitro* human CD4+CD25^hi+^ T_Reg_ and CD4+CD25^-^ responder T cells death/growth arrest by differentially using the granzyme-perforin pathway depending on its concentration. We observed a direct correlation between plasma E2 and IL-2 levels, compatible with what we have previously described of how E2 enhances *in vitro* the suppressive activity of CD4+ T_Reg_ mediated by perforin [17]. Taking this into account and considering the limitations of the small sample size, we can speculate that our *ex vivo* data suggest a circuit relating E2, IL-2, perforin and CD4+ T_Reg_. Also, serum IFN-α was higher in women in agreement with previously described [28], and here we show a direct correlation with serum E2 levels.

## 3. Experimental Section

### 3.1. Patients

A total of 26 patients: Males (*n*: 7) and females (*n*: 19) (median age: 38 years old, range: 21 to 57 years old) with first clinical isolated syndrome (CIS) that later on fulfilled MS diagnosis criteria defined by MCDonald’s criteria at first clinical relapse were enrolled in this study. One-hundred and nineteen sex-and-age matched healthy controls were enrolled in the study (102 females (*n* = 66 at day 1 and *n* = 36 at day 14 of the menstrual cycle) and 17 males) (median age: 34.5 years-old, range: 19 to 50 years). None of the MS patients had received any anti-inflammatory, immunosuppressive or disease-modifying agents at least 3-months before blood sampling for this study. Relapse was defined as the appearance or reappearance of one or more neurological abnormalities that persisted for at least 24 h and which had been preceded by at least 30 days of stable or improved neurological state, without any underlying infectious disease. Clinical physical severity was quantified by Kurtzke’s Expanded Disability Status Scale (EDSS).

The Hospital Review Board approved the study protocol and written informed consent was obtained from all participants prior to study commencement and sample collection.

### 3.2. Blood and CSF Sampling

Peripheral blood and 1 to 3 mL of CSF samples were obtained at the same time by venipuncture and lumbar puncture, respectively. We counted approximately between 130,000 to 200,000 lymphocytes per 3 mL of CSF in most MS patients. Both samples were processed within 2-h from collection. The study was conducted according to the ethical guidelines of our institution and the Declaration of Helsinki.

### 3.3. Characterization and Quantitative Analysis of Regulatory CD4+ T Lymphocytes

Peripheral blood samples were taken and collected in EDTA Vacutainers and processed immediately. Whole blood (100 μL) samples were labeled by direct staining with appropriate fluorochrome-conjugated monoclonal antibodies (mAbs) for 20 min at room temperature and then lysed and washed, as previously described [3]. CSF was processed immediately after spinal tap, centrifuged after collection and then the obtained cells were resuspended in 100 μL of complete medium (RPMI 1640 containing 10% foetal calf serum (FCS), streptomycin, penicillin and glutamine) before staining with the corresponding antibodies for 30 min at 4 °C in the dark. Direct conjugated mAbs: CD4-FITC, CD8-FITC, CD25 APC were obtained from Becton Dickinson (BD Immunocytometry Systems, San Jose, CA, USA).

**Figure 3:**
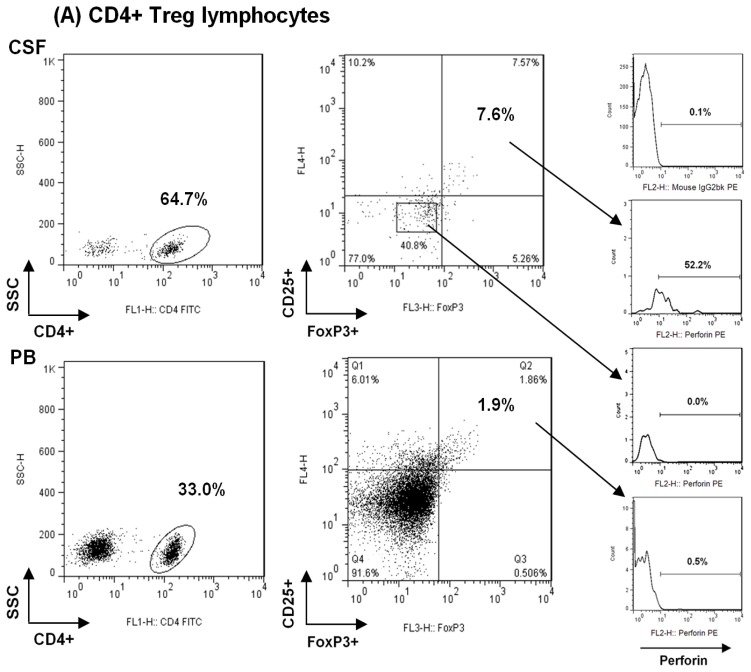

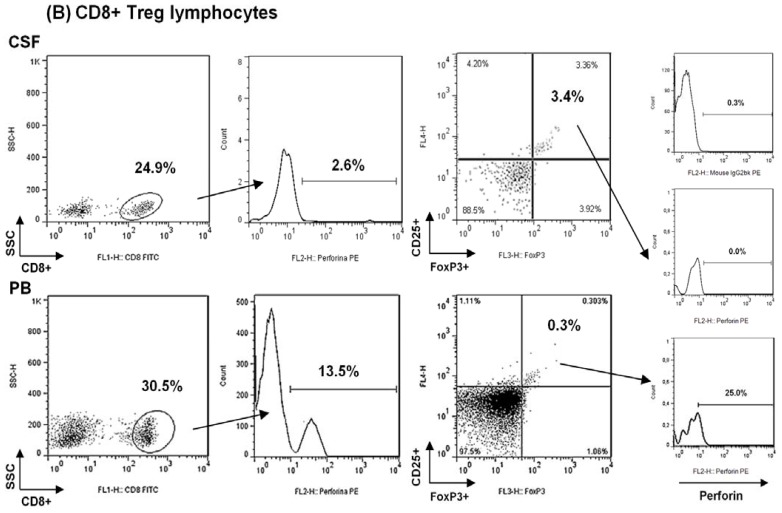
(**A**) shows the gating strategy by which CD4+T_Reg_ were characterized and their perforin staining; (**B**) shows CD8+ T_Reg_ gating strategy and perforin staining of CD8+ T cells as positive control for perforin staining. CD8+ T cells showed lower expression of perforin in the CSF than in the periphery.

T_Reg_ were defined as CD4+CD25+FoxP3+ and CD4+CD25^hi+^ co-expression, as previously described [3]. Expression of FoxP3 and perforin were assessed by intracellular staining (Figure 3). Briefly, previously surface-stained cells were resuspended in 1 mL of freshly prepared permeabilization solution (eBioscience Fixation/Permeabilization), incubated for 30 min at 4 °C in the dark and washed. Next, cells were stained with anti-human FoxP3 antibody (PE-Cy5, clone PCH101, eBioscience) and anti-human Perforin antibody (PE, clone δG9, BD Pharmingen) and after 30 min incubation at 4 °C, cells were washed. Isotype control mAbs were used to determine background fluorescence levels. Acquisition and analysis were performed in a FACSort flow cytometer (Becton Dickinson), using a CELLQUEST Software (Becton Dickinson). A gate was drawn to include the lymphocytes in a dot plot of forward scatter *vs*. side scatter and a total of 20,000 events were collected in peripheral blood and at least 3000 events in CSF.

The frequencies of these subsets are expressed as percentage of total CD4+ T-lymphocytes. In a subgroup of patients (*n* = 10), the mean fluorescence intensity (MFI) of CD28 on CD4+CD25^hi+^ cells was also analyzed.

#### Analysis of Regulatory CD8+ T Lymphocytes

For analysis of the regulatory CD8+ T lymphocytes, a first gate on a side scatter/C8-FITC dot plot was set up to collect CD8+ high cells. A second dot plot (FoxP3-PE-Cy5/CD25-APC) was set up to quantify CD8+ T_Reg_, as previously described [11]. Within the CD8+CD25+FoxP3+ T_Reg_ subset, the content of perforin was measured.

### 3.4. Sex Hormones and Cytokine Detection

Serum samples were obtained about 8:30 am from all patients and controls. We separated serum in a refrigerated centrifuge, and stored at −80 °C until use. Serum cortisol was quantified by competitive immunoassay (IMMULITE 2000, Diagnostic Products Corporation, Los Angeles, CA, USA). Serum testosterone, E2 and P2 were determined by a chemiluminescent immunoassay (Immuno I, Bayer, Leverkusen, Germany).

The following cytokines were measured in plasma and in CSF of each patient using the Human Basic Kit FlowCytomix (eBioscience), according to the manufacturer’s instructions: IL12p70, IFN-γ, IL17a, IL2, IL10, IFN-α, IL4, IL5, IL1b, TNF-α, RANTES.

### 3.5. Statistical Analysis

Data are presented as mean ± standard deviation (SD), median (interquartile range). To compare the percentage of expression of each T-cells subpopulation, we used the Mann-Whitney *U*-test to compare pairs of groups. Differences between groups were considered significant at *p* < 0.05. All statistical analyses were performed using the SPSS (SPSS, Inc, Chicago, IL, USA) software packages. The correlation between variables was analyzed by Spearman coefficient.

## 4. Conclusions

In summary, a recruitment of both CD4+ and CD8+ T_Reg_ was found within the CSF during MS relapse. Here, we describe that perforin expression by CD4+ T_Reg_, was greatly enhanced in CSF, suggesting a relevant role of this molecule in the cell-contact suppressive effects of the CD4+ T_Reg_ in MS. Moreover, the proportions of CD4+ T_Reg_ in the CSF were significantly higher in women than in men, which could account for the sexual dimorphism clinically evident in the incidence of MS. Despite the small sample size of our study, our preliminary data indicate a direct correlation between plasma E2 and IL-2 levels, which suggest that a putative circuit of E2 and perforin expression by CD4+ T_Reg_ play a role in MS pathophysiology. Further studies should be performed to confirm these findings.

## Figures and Tables

**Table 1:** Proportions of regulatory CD8+ T-cells subsets in cerebrospinal fluid (CSF) and peripheral blood (PB) in patients at first MS relapse.

	CSF	PB	*P*
**% CD8+FoxP3+ T cells**	0.95 ± 1.00 0.99 (1.49)	0.13 ± 0.08 0.14 (0.12)	0.02
**% CD8+CD25+FoxP3+ T cells**	0.84 ± 1.05 0.60 (1.23)	0.13 ± 0.07 0.16 (0.15)	0.02
